# Cannabinoids drive Th17 cell differentiation in patients with rheumatic autoimmune diseases

**DOI:** 10.1038/s41423-020-0437-4

**Published:** 2020-04-28

**Authors:** Konstantin Kotschenreuther, Iris Waqué, Shuaifeng Yan, Anja Meyer, Thom Haak, Julia von Tresckow, Joanna Schiller, Lydia Gloyer, Mara Dittrich-Salamon, David M. Kofler

**Affiliations:** 1 Laboratory of Molecular Immunology and Division of Clinical Immunology and Rheumatology, University of Cologne, Cologne, Germany; 2 Department I of Internal Medicine, University of Cologne, Cologne, Germany

**Keywords:** CD4-positive T cells, Autoimmunity, Cytokines, Lymphocyte differentiation

The legalization of cannabinoids for medical use has reinforced their emerging role as a treatment of chronic pain in patients with cancer or rheumatic diseases.^1,2^ In addition to their role as pain relievers, evidence obtained from animal models suggests that cannabinoids have immunosuppressive properties.^3^ However, a definite immunosuppressive function of cannabinoids has not yet been confirmed in clinical trials.^4^ We therefore analyzed the influence of the cannabis derivative cannabidiol (CBD) and the endogenous cannabinoid anandamide (AEA) on T helper type 17 (Th17) cells from patients with rheumatoid arthritis (RA), systemic lupus erythematosus (SLE), and psoriatic arthritis (PsA). Interestingly, in vitro culture in the presence of CBD significantly increased Th17 cell differentiation in CD4+ T cells from the peripheral blood of patients with RA, SLE, or PsA, while Th17 cell differentiation was suppressed in healthy individuals (Fig. 1a and Supplementary Fig. S1A). In RA patients, the median Th17 cell frequency in CBD-treated cells was 6.54 ± 0.52 vs. 3.27 ± 0.23 in the vehicle control group (*p* < 0.0001), and in healthy controls, the frequency was 1.86 ± 0.25 in CBD-treated cells vs. 3.62 ± 0.32 in the vehicle control group (*p* = 0.0002). AEA showed similar effects on CD4+ T cells from patients but did not affect CD4+ T cells from healthy controls (Fig. 1a). The addition of the Th17 skewing cytokines transforming growth factor-β, interleukin (IL)-1β, IL-6, and IL-23 further increased the Th17-inducing properties of CBD (Fig. 1b). As shown previously in experimental autoimmune encephalomyelitis (EAE) mice, the production of interferon-γ and tumor necrosis factor-α was reduced in the presence of CBD in patients with rheumatic diseases, as well as in healthy individuals (Supplementary Fig. S1B, C). During our study, some of our RA patients reported the use or planned use of CBD oil as a pain reliever. In these cases, we compared Th17 cell frequencies before and after treatment initialization and found that treatment with CBD oil for 4–8 weeks drove Th17 cell expansion in vivo (1.10 ± 0.32 before vs. 4.52 ± 1.34 after CBD treatment; Fig. 1c). Interestingly, disease activity measured by Disease Activity Score 28-joint count C reactive protein significantly increased during CBD treatment (Fig. 1d). In accordance with previous reports, this immunomodulatory effect of CBD was not mediated by the receptors CB1, CB2, or GPR55 (Supplementary Fig. S2A).^5^ To further assess the characteristics of the CBD-induced Th17 cells, we analyzed their gene expression profiles and discovered a CBD-mediated increase in *SGK1* expression (Fig. 1e, Supplementary Fig. S2C). This is remarkable, as SGK1 is an important regulator of the reciprocal development of proinflammatory Th17 cells.^6^ In addition, the expression of *CSF2* was decreased and the expression of *AHR* was increased by CBD (Fig. 1f–g  and Supplementary Fig. S2B, C).

**Fig. 1:**
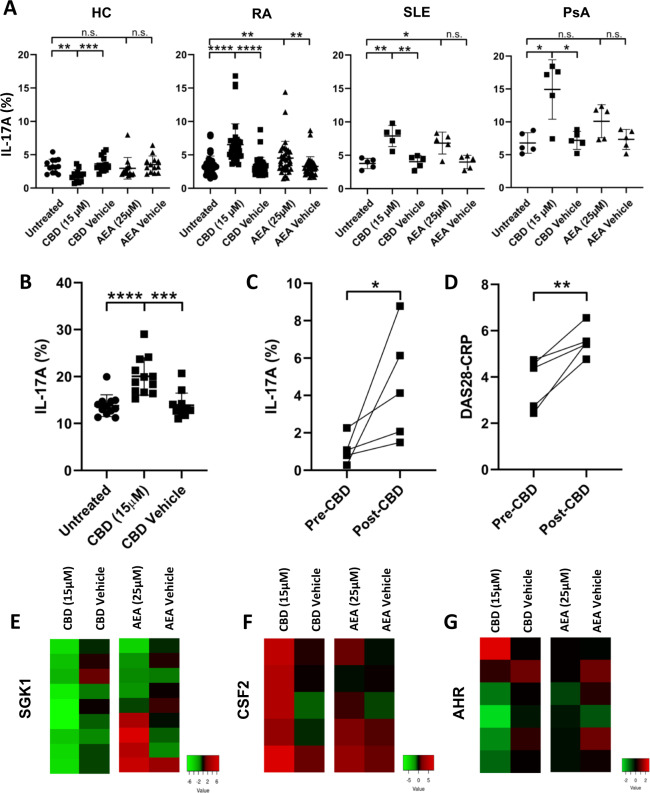
Cannabinoids induce Th17 cell differentiation in patients with rheumatic diseases. **a** Expression of IL-17A in CD4+ T cells from healthy controls and patients was analyzed by flow cytometry (HC, *n* = 13; RA, *n* = 36; SLE, *n* = 5, PsA, *n* = 5). **b** CBD-mediated induction of IL-17A expression in CD4+ T cells from RA patients in the presence of TGFβ, IL-1β, IL-6, and IL-23 (*n* = 12). **c** Increase in IL-17A-positive Th17 cells in patients receiving cannabidiol oil for 4–8 weeks (*n* = 5). **d** DAS28-CRP in patients receiving cannabidiol oil for 4–8 weeks (*n* = 5). **e**–**g** Heat maps showing gene expression in RA patients treated with CBD or AEA. Gene expression was analyzed by RT-PCR. *p < 0.05, **p < 0.01, ***p < 0.001, ****p < 0.0001; the data are presented as the mean ± SEM; significant differences were determined using the unpaired Mann–Whitney test and Student’s *t* test

Th17 cells play a central role in the pathogenesis of PsA and ankylosing spondylitis. In addition, they have been linked at least partly to the pathogenesis of various other rheumatic autoimmune diseases. We observed an increase in Th17 cell frequencies induced by CBD in vitro, as well as in some patients with RA receiving CBD treatment. These results are in contrast to observations made in mice with EAE, in which cannabinoids ameliorated disease activity.^3^ However, CB2-selective agonists are often used in these animal studies.^3^ The CB2 receptor is known to mediate immunosuppressive effects, while immune-activating effects have been attributed to other receptors.^3^ We used cannabinoids that activate various receptors and pathways. Variations in these receptors and pathways between patients with rheumatic autoimmune diseases and healthy individuals could explain differences between patients and healthy subjects. Moreover, the variety of CBD receptors could be responsible for the discrepancy between animal studies and findings in humans, including our study. In conclusion, our data show that cannabinoids increase Th17 cell frequencies and suggest that they may therefore be used with caution in patients with rheumatic autoimmune diseases.

## Supplementary information

Supplementary Table S1

Supplementary Methods

Supplementary Figure S1

Supplementary Figure S1

Supplementary Figure S2

Supplementary Figure S2
