# Supplementary material for: Cannabinoids drive Th17 cell differentiation in patients with rheumatic autoimmune diseases
Source: Cell Mol Immunol. 2020 Apr 28;18(3):764–6. doi: 10.1038/s41423-020-0437-4 (PMC8027621; doi:10.1038/s41423-020-0437-4)
Supplement: Supplementary file 1 — Supplementary Table S1 [file 41423_2020_437_MOESM1_ESM.docx]

| **Patient characteristics** | **average** | **range** |
| --- | --- | --- |
| Age (years) | 58 | 31-84 |
| Sex ( f : m ) | 3.8:1 |  |
| Time since disease onset (years) | 11.14 | 0 - 39 |
| Number of previous treatments | 3.62 | 1-10 |
| DAS-28(BSG) | 2.85 | 1.11 – 6.05 |
| DAS-28 (CRP) | 2.58 | 0.96 - 6 |
| CRP [mg/l] | 6.12 | 06 – 62.7 |
| BSG [mm/h] | 16 | 2 - 71 |
| Rheumatoid factor (% positive) | 51 |  |
| ACPA (% positive) | 56 |  |

**Supplementary Table S1: Characteristics of patients with RA**
