# Supplementary material for: Cannabinoids drive Th17 cell differentiation in patients with rheumatic autoimmune diseases
Source: Cell Mol Immunol. 2020 Apr 28;18(3):764–6. doi: 10.1038/s41423-020-0437-4 (PMC8027621; doi:10.1038/s41423-020-0437-4)
Supplement: Supplementary file 2 — Supplementary Methods [file 41423_2020_437_MOESM2_ESM.docx]

**Supplementary Methods**

*Study subjects.* All patients with RA fulfilled the 2010 ACR/EULAR classification criteria. Blood samples from age- and sex-matched healthy individuals were used as controls. Blood was drawn after written informed consent was obtained in accordance with the Declaration of Helsinki following a study protocol approved by the Ethics Committee of the University of Cologne (4).

*Human T cell isolation.* Primary human lymphocytes were isolated from peripheral blood from patients and healthy controls by Pancoll® density gradient centrifugation (PAN™-Biotech GmbH, Aidenbach, Germany). CD4^+^ T cells were purified by negative selection using the CD4⁺ T cell isolation Kit. The purity of the CD4+ T cell populations was verified by flow cytometry and was at least 96%. Viable cells were counted using the Vi-CELL XR cell counter (Beckman Coulter, Krefeld, Germany) or the automated cell counter CellCountess (Life Technologies GmbH, Darmstadt, Germany).

*Th17 polarization.* CD45RA^+^RO⁻ naïve CD4^+^ T cells were cultured in X-Vivo 15 (Lonza, Cologne, Germany) media supplemented with 1% human serum and 1% Penicillin-Streptomycin (both Sigma-Aldrich, Saint Louis, U.S.). Cells were cultured for 4 days with the T cell Activation/Expansion Kit (Miltenyi Biotec) and recombinant human TGF-β (5 ng/µl; PAN™-Biotech GmbH, Aidenbach, Germany), IL‑1β (12.5 ng/µl), IL-6 (25 ng/µl; both Miltenyi Biotec) and IL-23 (25 ng/µl; PeproTech, Rocky Hill, U.S.). Medium and cytokines were refreshed after 3 days.

*Flow cytometry.* CD4^+^ T cells were stimulated with PMA (100 ng/ml) and ionomycin (1.5 µM; both Cell Signaling Technology®, Danvers, U.S.) in the presence of Brefeldin A (eBioscience, San Diego, U.S.) for 3 hours. To exclude dead cells, cells were stained by the LIVE/DEAD™ Fixable Dead Cell Stain Kit (Invitrogen, ThermoFisher Scientific, Carlsbad, U.S.). For intracellular staining, cells were fixed and made permeable by the BD Cytofix/Cytoperm Kit (BD Bioscience, Heidelberg, Germany) according to the manufacturer´s instructions and stained with anti-IL17A, anti-IFN-γ (both affymetrix eBioscience). Data were acquired on the Gallios 10/3 flow cytometer (Beckman Coulter, Krefeld, Germany).

*Quantitative Real Time PCR.* RNA was isolated from CD45RA^+^RO^-^ T cells using the RNeasy Mini Kit and converted into cDNA using the QuantiTectReverse Transcription Kit (both Qiagen, Hilden, Germany). All primers were purchased from Applied Biosystems. All reactions were performed using the 7500 Fast Real-Time PCR System (Applied Biosystems). The values are represented as the difference in C_t_ values normalized to β2‑microglobulin for each sample using the following formula: relative RNA expression = (2‑^dCt^) x 10^3^.

*Statistics.* Statistical analysis was performed using GraphPad Prism. Where indicated, data were analyzed by non-parametric Mann-Whitney test or student’s t-test and are presented as the mean ± SEM. *p*< 0.05 was considered as statistically significant.
