# Supplementary figures and images for: Cannabinoids drive Th17 cell differentiation in patients with rheumatic autoimmune diseases
Source: Cell Mol Immunol. 2020 Apr 28;18(3):764–6. doi: 10.1038/s41423-020-0437-4 (PMC8027621; doi:10.1038/s41423-020-0437-4)

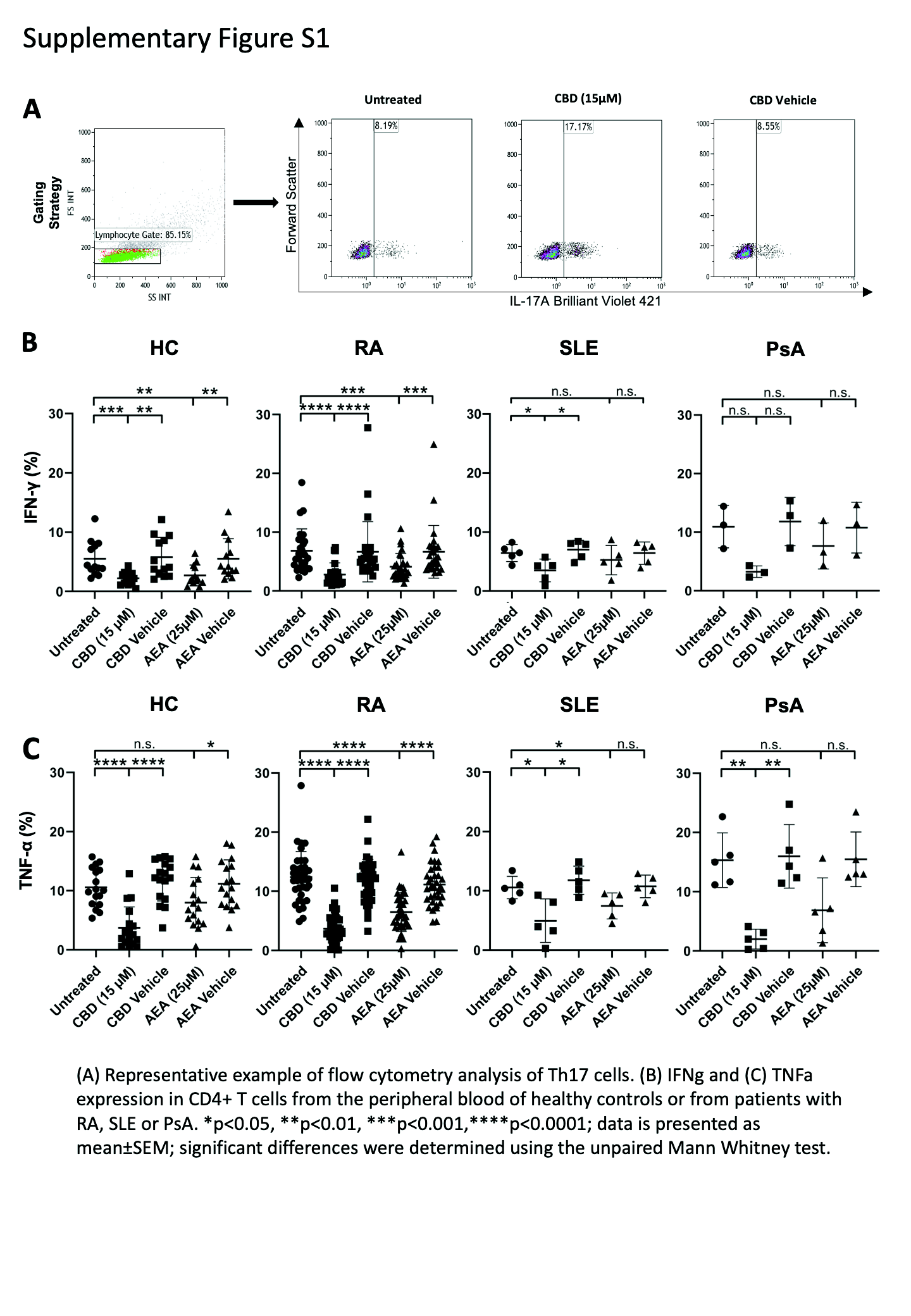

Supplement: Supplementary file 3 — Supplementary Figure S1 [file 41423_2020_437_MOESM3_ESM.tif]

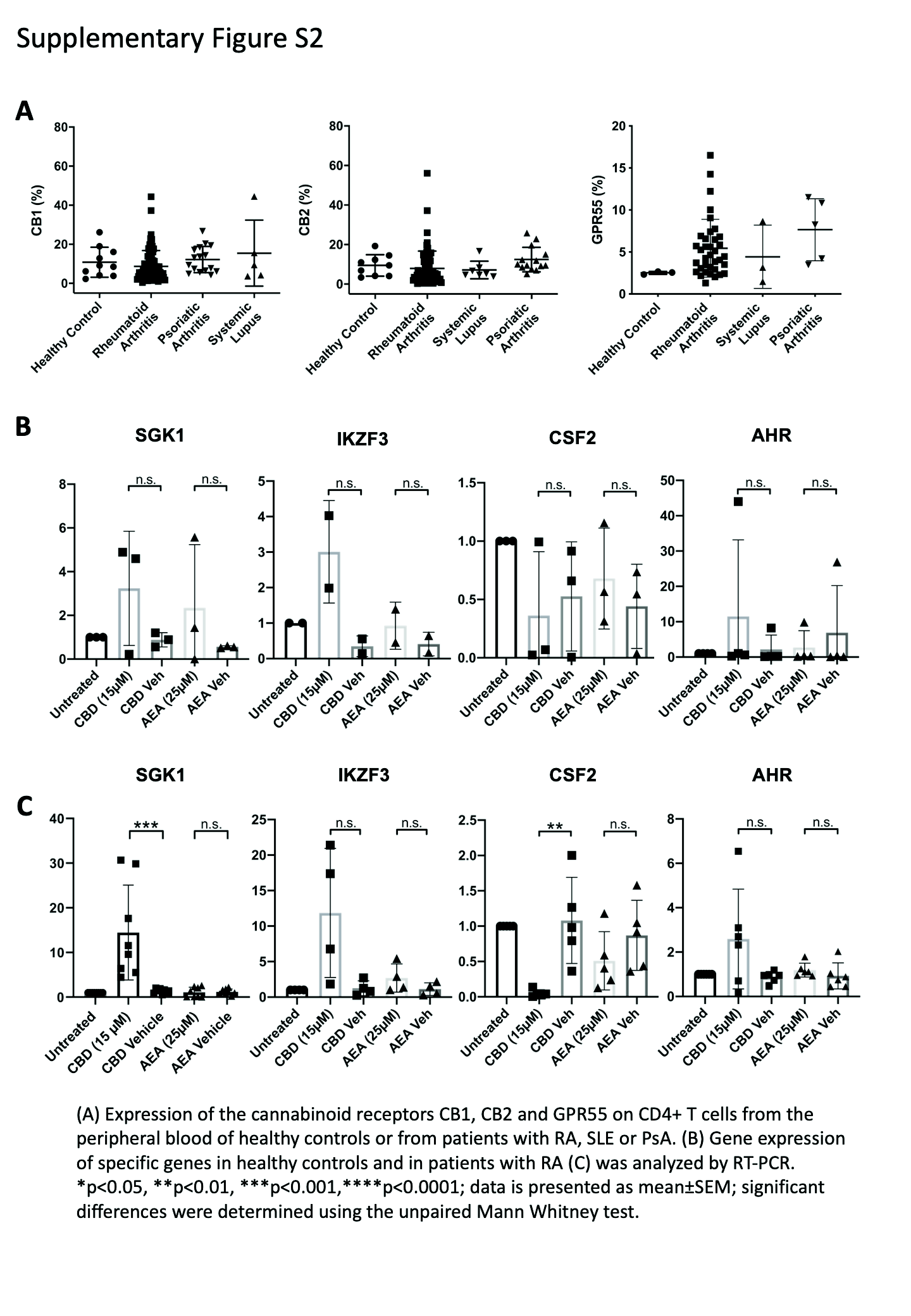

Supplement: Supplementary file 5 — Supplementary Figure S2 [file 41423_2020_437_MOESM5_ESM.tif]
