# Supplementary material for: Cannabinoids drive Th17 cell differentiation in patients with rheumatic autoimmune diseases
Source: Cell Mol Immunol. 2020 Apr 28;18(3):764–6. doi: 10.1038/s41423-020-0437-4 (PMC8027621; doi:10.1038/s41423-020-0437-4)
Supplement: Supplementary file 4 — Supplementary Figure S1 [file 41423_2020_437_MOESM4_ESM.pptx]

## Slide 1
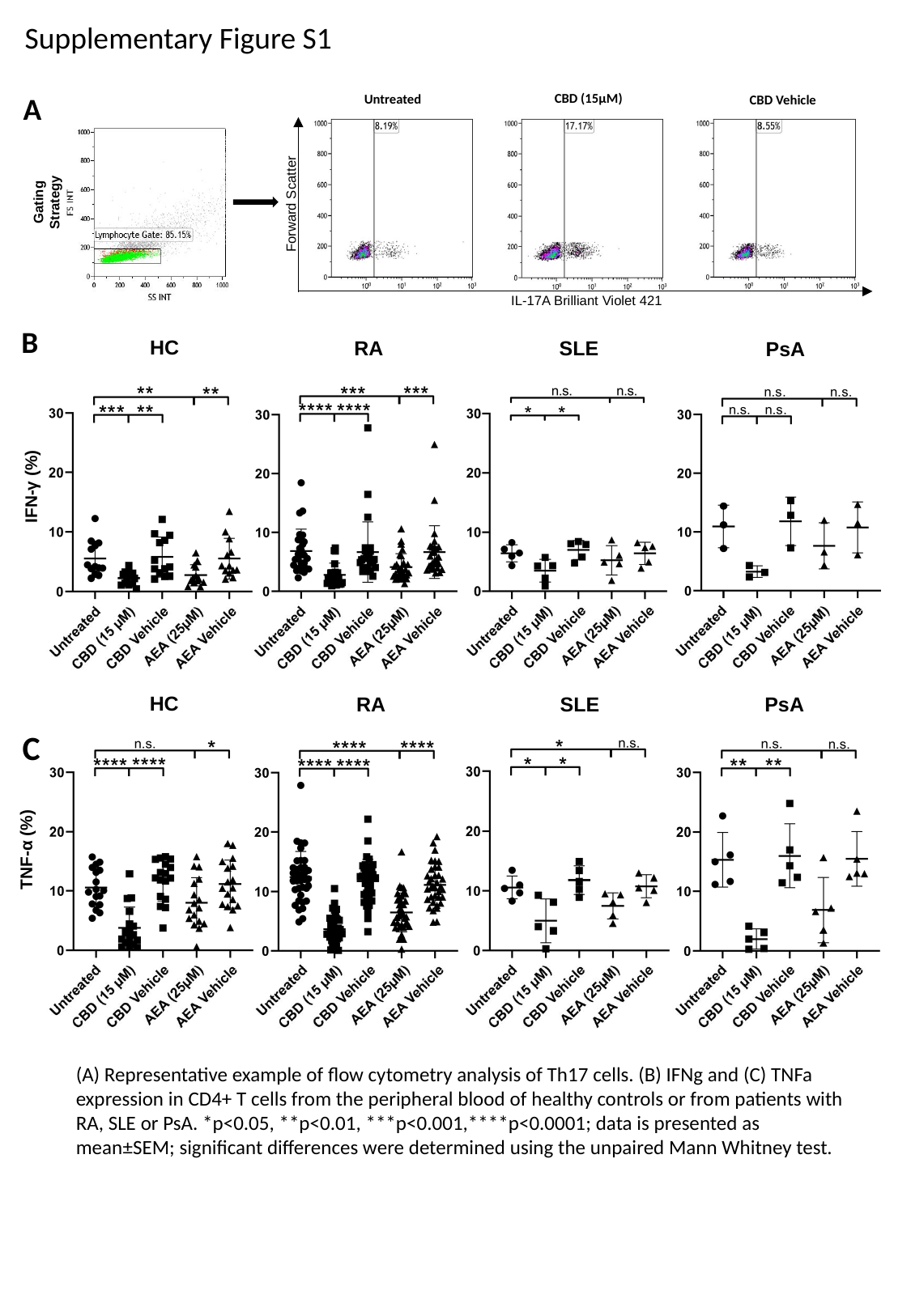

Supplementary Figure S1
CBD (15µM)
Untreated
CBD Vehicle
Forward Scatter
IL-17A Brilliant Violet 421
A
Gating Strategy
B
HC
SLE
RA
PsA
 IFN-γ (%)
HC
SLE
RA
PsA
C
TNF-α (%)
(A) Representative example of flow cytometry analysis of Th17 cells. (B) IFNg and (C) TNFa expression in CD4+ T cells from the peripheral blood of healthy controls or from patients with RA, SLE or PsA. *p<0.05, **p<0.01, ***p<0.001,****p<0.0001; data is presented as mean±SEM; significant differences were determined using the unpaired Mann Whitney test.
