# Supplementary material for: Cannabinoids drive Th17 cell differentiation in patients with rheumatic autoimmune diseases
Source: Cell Mol Immunol. 2020 Apr 28;18(3):764–6. doi: 10.1038/s41423-020-0437-4 (PMC8027621; doi:10.1038/s41423-020-0437-4)
Supplement: Supplementary file 6 — Supplementary Figure S2 [file 41423_2020_437_MOESM6_ESM.pptx]

## Slide 1
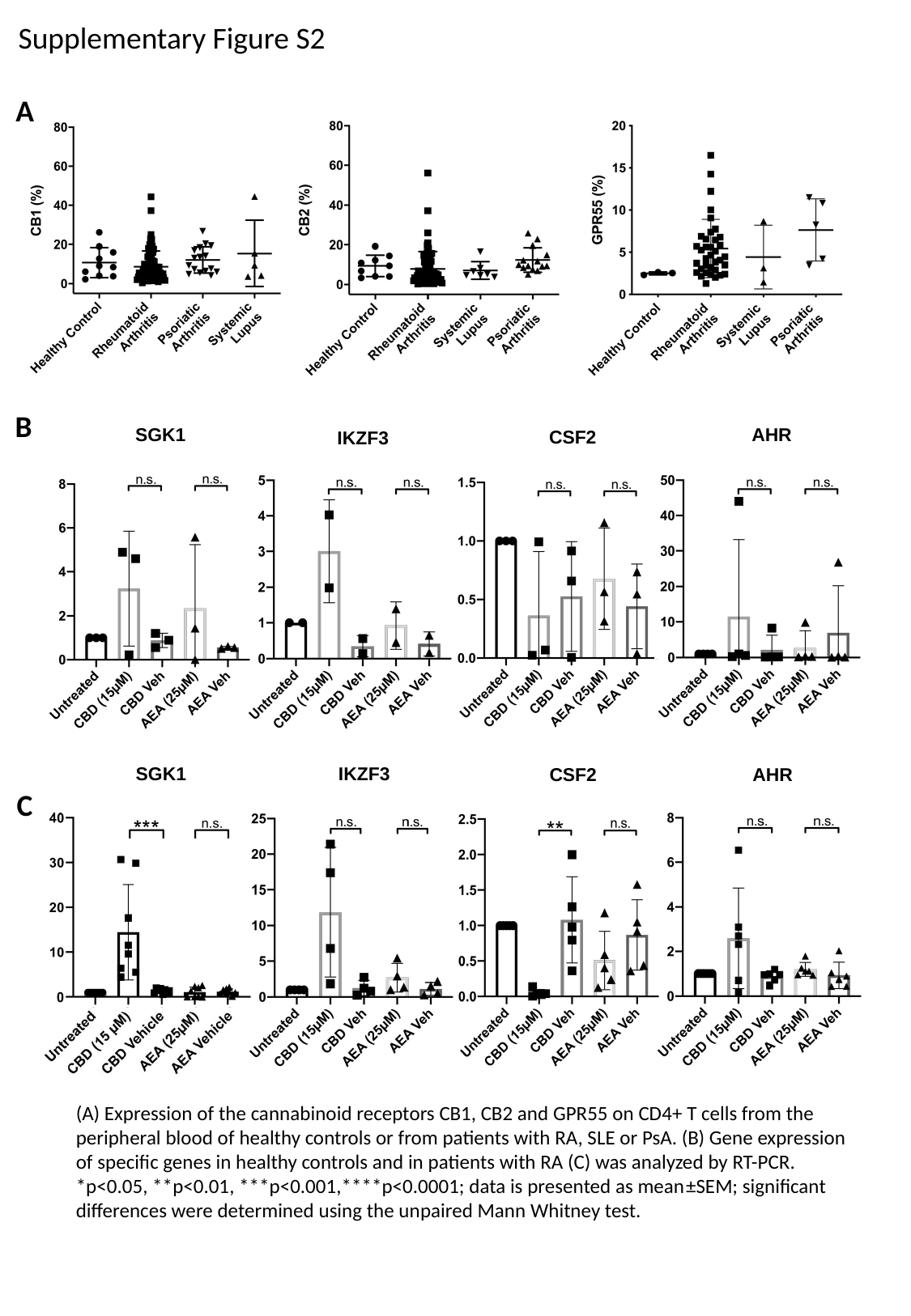

Supplementary Figure S2
A
B
SGK1
AHR
CSF2
IKZF3
SGK1
IKZF3
CSF2
AHR
C
(A) Expression of the cannabinoid receptors CB1, CB2 and GPR55 on CD4+ T cells from the peripheral blood of healthy controls or from patients with RA, SLE or PsA. (B) Gene expression of specific genes in healthy controls and in patients with RA (C) was analyzed by RT-PCR. *p<0.05, **p<0.01, ***p<0.001,****p<0.0001; data is presented as mean±SEM; significant differences were determined using the unpaired Mann Whitney test.
